# Association between risk of brucellosis and genetic variations in MicroRNA-146a

**DOI:** 10.1186/s12879-021-06775-4

**Published:** 2021-10-16

**Authors:** Sima Kazemi, Saeid Afshar, Manoochehr Karami, Massoud Saidijam, Fariba Keramat, Seyed Hamid Hashemi, Mohammad Yousef Alikhani

**Affiliations:** 1 Department of Microbiology, Faculty of Medicine, Hamadan University of Medical Sciences, Hamadan, Iran; 2 Research Center for Molecular Medicine, School of Medicine, Hamadan University of Medical Sciences, Hamadan, Iran; 3 Department of Biostatistics and Epidemiology, School of Public Health, Hamadan University of Medical Sciences, Hamadan, Iran; 4 Brucellosis Research Center, Hamadan University of Medical Sciences, Hamadan, Iran

**Keywords:** Brucellosis, Genetic variations, miR-146a

## Abstract

**Background:**

Single nucleotide polymorphisms (SNPs) are the most common types of DNA changes in the human genome that leading to phenotypic differences in humans. MicroRNAs (miRNAs) are usually affected by various bacterial infections, and they are involved in controlling the immune responses. MicroRNA-146a (miR-146a) plays an essential role in the development of infectious and inflammatory diseases. The aim of the present study was to investigate the association between risk of brucellosis and genetic variations in miR-146a.

**Methods:**

This case–control study was conducted on 108 Brucellosis patients and 108 healthy controls. We genotyped two SNPs (rs2910164 and rs57095329) of the miR-146a using tetra-primer amplification refractory mutation system-polymerase chain reaction (T-ARMS-PCR) and restriction fragment length polymorphism-polymerase chain reaction (RFLP-PCR) methods.

**Results:**

The rs2910164 SNP was significantly associated with brucellosis in co-dominant [OR = 4.27, 95% CI = (2.35–7.79, *P* = 0.001] and dominant [OR = 3.52, 95% CI = (1.97–6.30, *P* = 0.001] models. Co-dominant (*P* = 0.047) and recessive (*P* = 0.018) models were significant at position rs57095329 between the two groups of patient and healthy. The A C haplotype (rs2910164 and rs57095329) was associated with brucellosis in the assessed population [OR (95% CI) = 1.98 (1.22–3.20), *P* = 0.0059].

**Conclusions:**

Consequently, our study demonstrated significant differences in genotype and haplotype frequencies of miR-146a variants between brucellosis patients and controls. Further studies on the larger sample sizes are required to verify the observed associations.

## Background

One of the highly noticeable zoonotic diseases globally is brucellosis, particularly in developing countries caused by the genus *Brucella* [1–3]. Bacteria are transmitted to humans by consumption of unpasteurized dairy products, direct contact with the various components of the infected animal, or inhalation of aerosols [4]. The prevalence of human diseases in endemic areas is more than 10 per 100,000 population. Hamadan province, in the western part of Iran, has the highest incidence in the country, with 130 cases per 100,000 population [5]. Brucellosis causes considerable economic losses in numerous countries every year by damage to livestock via reduced fertility, abortion, and reduction in meat production, and milk [6]. MicroRNAs (miRNAs) are a type of evolutionary conserved small single-stranded non-coding RNAs which control the target gene expression negatively via mRNA degradation or translational expression at the post transcriptional level [7–9]. Furthermore, a miRNA is capable of modulating the expression of several mRNAs, and one mRNA can be targeted by many miRNAs [10]. MiRNAs play a crucial regulatory role in extensive types of biological processes such as cell differentiation, early evolution, autophagy, metabolism, apoptosis, proliferation, DNA repair, and immune responses [10–12]. MicroRNAs have also been demonstrated to have a major role during infection by diverse pathogens, including viruses, parasites, and bacteria [13–15]. Additionally, the potential importance of miRNAs in the immune response to intracellular and extracellular pathogens such as *Mycobacteria* spp., *Helicobacter pylori*, *Salmonella enterica, Listeria monocytogenes*, *Staphylococcus aureus*, *Pseudomonas aeruginosa*, *Citrobacter rodentium*, *Francisella* spp., *chlamydia* spp., *Burkholderia pseudomallei* and *Shigella flexneri* has been demonstrated [16–25]. Control of miRNAs has been identified as a key regulatory factor in the immune system [26]. Single nucleotide polymorphisms (SNPs) represent the most abundant genetic variation in the entire human genome [27]. Researches have shown that genetic alterations in miRNA sequences can affect primary miRNA (pri-miRNA) and precursor miRNA (pre-miRNA) processing and thus alter miRNA expression [28]. Following changes in the polymorphisms of these molecules, they may have possible consequences for the regulation of gene expression, and the progress of the diseases [29]. MiR-146a, is one of the highly conserved miRNAs, which is recognized for its crucial balancing of the inflammation and immune responses [30], and bacterial infections, especially mycobacteria [31–33]. Rs2910164 and rs57095329 are the two most remarkable and widely seen SNP in miR-146a. They can affect the level of mature miR-146a and are related to different types of cancer, many inflammation-associated diseases, and important neurological and infectious diseases such as systemic lupus erythematosus (SLE), Behcet’s disease, sepsis, rheumatoid arthritis, tuberculosis (TB), hepatitis, Alzheimer’s disease (AD), and multiple sclerosis [33–41]. The miR-146a polymorphism rs2910164 is located on chromosome 5q33 [42]. Polymorphism rs2910164 (G > C) in pre-miR146a, leads to a change from a G:U pair to a C:U mismatch in its stem region [40, 43]. The A/G polymorphism of rs57095329 in the miR-146a is specified to be located in the promoter region [36]. It is also demonstrated that the level of mature miR-146a is reduced by lowering the transcription factor binding Vets oncogene-homolog-1 which was related to the risk of SLE [36]. MiR-146a plays a pivotal role in regulating the immune responses. Therefore, there may be an association between the SNP molecule and the immune responses to pathogens and the risk of infectious diseases like brucellosis [44]. To date, there are no data showing the relevance between miR-146a polymorphism with brucellosis disease. Therefore, we conducted an evaluation study to find the relationship between polymorphisms of miR-146a (rs57095329 and rs2910164) with brucellosis patients as well as healthy controls in Hamadan province’s population, the west of Iran.

## Methods

### Study subjects

This case–control study included 108 brucellosis patients and 108 controls recruited from the Farshchian’s Hospital of Hamadan between February 2018 and August 2019. Diagnosis of the disease was confirmed according to the following criteria: (1) clinical manifestations such as arthralgia, weight loss, malaise, fever, hepatomegaly, focal complication, splenomegaly, and sweating (2) detection of specific antibody titers in the serum samples of individuals who referred to the Infectious Diseases Ward, Farshchian’s Hospital, using Wright titer ≥ 1/160 and 2-mercaptoethanol test (2ME) ≥ 1/80, and 3) isolation of *Brucella spp.* in blood, using a BACTEC blood culture system (9050 BD Company, U.S.A.), which is a fully automated microbiology growth and detection system designed to detect microbial growth from blood specimens or PCR method to identify the *Brucella* isolates, targeting the *bcsp31* gene coding a 31-kDa outer membrane protein *Brucella spp*.[45]. Exclusion criteria in this study were inflammatory diseases including cardiovascular diseases, severe hematological, gastrointestinal, neurological diseases, patients with the deficiency of glucose 6-phosphate dehydrogenase (G6PD), or pregnant women or people with diseases such as psoriasis and porphyria [46].

The control group consisted of individuals without brucellosis, also evaluated with any antibody to brucellosis in serological tests. Healthy subjects were selected from the same geographical location of the patients. The study protocol was approved by the Ethics Committee of Hamadan University of Medical Sciences, Hamadan, Iran (Ethical committee ID: IR.UMSHA.REC.1397. 857).

### DNA extraction

Genomic DNA was extracted from peripheral whole blood samples from patients and controls by the salting out technique [47]. All DNA samples were dissolved in water and stored at − 20 °C for future use.

### Genotyping

SNPs of the miR-146a gene (rs2910164 G/C and rs57095329 A/G) were assessed by tetra-primer amplification refractory mutation system-polymerase chain reaction (T-ARMS-PCR) and restriction fragment length polymorphism-polymerase chain reaction (RFLP-PCR), respectively. The primers and PCR conditions used for the mir-146a (rs2910164 G/C) are as follows: FO (Outer): 5′GGCCTGGTCTCCTCCAGATGTTTAT3′, RO: 5′ATACCTTCAGAGCCTGAGACTCTGCC3′, FI (Inner) (C allele): 5′ATGGGTTGTGTCAGTGTCAGACGTC3′, RI (Inner) (G allele): 5′GATATCCCAGCTGAAGAACTGAATTTGAC 3′ [48]. The PCR assay was performed using commercially available PCR premix (PCR 2X Taq premix Mastermix; (Ampliqon, Denmark). Briefly, ~ 100 ng/μL templates DNA, 1 μL of each primer (10 pmol/μL) were added to PCR PreMix at a final volume of 20 μL. The thermocycling conditions for rs2910164 G/C: 5 min at 95 °C, followed by 30 cycles of 30 s at 95 °C, annealing temperatures at 62 °C for 30 s, extension at 72 °C for 30 s and a final extension step in 72 °C for 10 min [48]. PCR products were separated by electrophoresis on 2% agarose gel. The primers used in the RFLP-PCR reaction (rs57095329 A/G) included 5′-GGGGCTGCGGAGAGTACCG-3′ and 5′-GGACCCTCTTGCAGCACGTGTC-3′ [49] and the restriction enzyme was MspI (Fermentase, Lithuania). PCR program for (rs57095329 A/G) was as follow: Initial denaturalization at 95 °C for 5 min, followed by 30 cycles of denaturation at 95 °C, 30 s, annealing for 30 s at 60 °C, extension at 72 °C for 30 s and a final extension step at 72 °C for 5 min. The digestion products were identified by silver staining [50]. Finally, 10% of the total samples randomly chosen to validate were sent for sequencing.

### Statistical analysis

Statistical assessment was performed using version 16 of SPSS. Even though, the Hardy–Weinberg equilibrium (HWE) was not maintained in both SNPs, the Expectation–Maximization (EM) algorithm was used in R software to calculate the frequency of alleles. The linkage disequilibrium (LD) between rs2910164 and rs57095329 were appraised by measurement of D’ and r values. Associations between the brucellosis and the rs2910164/ rs57095329 SNP were addressed by calculating odds ratios (ORs) and 95% confidence intervals (CI) in haplotype as well as in alleles, recessive, dominant, and co-dominant inheritance models. *P values* less than 0.05 were regarded as significant.

## Results

### Characteristics of the study population

The demographic characteristics of patients and control individuals are shown in Table 1. In this study consisted of 108 brucellosis patients (73 men and 35 women), (age range 15–87 years and mean ± SD = 15.19 ± 44.55) and 108 healthy subjects as a control group (85 men and 23 women), (age range 19–65 years and mean ± SD = 10.448 ± 37.38). There was a significant difference in age in both patient and healthy groups *(P* < 0.001). No significant difference in sex was observed between the two groups (*P* = 0.091). Besides, there was a significant difference in the habitat, consumption history of unpasteurized dairy products, history of contact with livestock, history of brucellosis and history of brucellosis treatment between patient, and control groups. (All *P* < 0.001).

**Table 1:** Demographic characteristics of brucellosis patients and control subjects

Factors	Case group n = 108	Control group n = 108	*P-value*
Age			
Mean ± SD	15.19 ± 44.55	10.448 ± 37.38	< 0.001
Range (years)	15–87	19–65	

	No (%)	No (%)	
Sex			
Men	73 (67.6)	85 (78.7)	0.091
Women	35 (32.4)	23 (21.3)	
Habitat			
Urban	17 (15.7)	(71.3) 77	< 0.001
Rural	91 (84.3)	31 (28.7)	
History of non-pasteurized dairy products			
Yes	87 (80.6)	–	< 0.001
No	21 (80.6)	108 (100)	
History of contact with livestock			
Yes	89 (82.4)	–	< 0.001
No	19 (17.6)	108 (100)	
History of Brucellosis			
Yes	29 (26.9)	–	< 0.001
No	79 (73.1)	108 (100)	
History of brucellosis treatment			
Yes	28 (25.9)	–	< 0.001
No	80 (74.1)	108 (100)	

### Association of single nucleotide polymorphisms in miR-146a with risk of brucellosis

The genotypes and alleles analyses of miR-146a SNP in patient and control subjects are shown in Table 2. At position rs2910164, the frequency of GC genotypes was higher in the control group compared to the patient group, while the frequency of genotype GG was higher in the patient group than in the control group. The rs2910164, co-dominant [OR = 4.27, 95% CI = 2.35–7.79, P = 0.001] and dominant [OR = 3.52, 95% CI = 1.97–6.30, P = 0.001] models were statistically significantly different between patients and controls. The rs57095329, co-dominant [OR = 0.63, 95% CI = 0.17–2.29, *P* = 0.047] and recessive (*P* = 0.018) models were significantly different between the patient and healthy groups. Analysis of alleles frequency of rs2910164 and rs57095329 revealed no statistically significant differences between patients and controls.

**Table 2:** Genotype and allele frequencies distribution in patients with brucellosis and controls

Model	Genotype	Cases (n = 108) No. (%)	Controls (n = 108) No. (%)	OR (95% CI)	*P-value*
MiR-146a rs2910164					
Allele	C	31 (28.7)	43 (39.8)	1	0.085
	G	77 (71.3)	65 (60.2)	1.64 (0.93–2.89)	
Co-dominant	GG	57 (52.8)	26 (24.1)	1.00	< 0.0001
	GC	40 (37)	78 (72.2)	4.27 (2.35–7.79)	
	CC	11 (10.2)	4 (3.7)	0.80 (0.23–2.74)	
Dominant	G/G	57 (52.8)	26 (24.1)	1.00	< 0.0001
	GC-CC	51 (47.2)	82 (75.9)	3.52 (1.97–6.30)	
Recessive	GG-GC	97 (89.8)	104 (96.3)	1.00	0.056
	CC	11 (10.2)	4 (3.7)	0.34 (0.10–1.10)	
MiR-146a rs57095329					
Allele	A	104 (96.3)	100 (92.6)	1	0.235
	G	4 (3.7)	8 (7.4)	2.08 (0.60–7.125)	
Co-dominant	AA	98 (90.7)	104 (96.3	1.00	0.047
	AG	6 (5.6)	4 (3.7)	0.63 (0.17–2.29)	
	GG	4 (3.7)	0 (0)	0.00 (0.00–NA)	
Dominant	AA	98 (90.7)	104 (96.3)	1.00	0.092
	AG-GG	10 (9.3)	4 (3.7)	0.38 (0.11–1.24)	
Recessive	AA-AG	104 (96.3)	108 (100)	1.00	0.018
	GG	4 (3.7)	0 (0)	0.00 (0.00–NA)	

### Haplotype analysis

The rs2910164 and rs57095329 SNPs were not in LD in the assessed population (D’ = 0.41, r = − 0.06). The A C haplotype (rs2910164 and rs57095329) increased the risk of brucellosis in the assessed population [OR (95% CI) = 1.98 (1.22–3.20), *P* = 0.0059]. Table 3 demonstrates the haplotypes in cases and controls.

**Table 3:** Haplotypes of rs2910164 and rs57095329 SNP in patients with brucellosis and controls

rs2910164	rs57095329	Patients %	Controls %	OR (95% CI)	*P-value*
G	A	66.08	58.79	1.00	–
C	A	27.44	39.35	1.98 (1.22–3.20)	0.0059
G	G	5.21	1.39	0.57 (0.19–1.67)	0.3
C	G	1.27	0.46		1

Global haplotype association *P-value*: 0.0069

## Discussion

In the current study, we examined the effects of two polymorphisms in miR-146a among patients with brucellosis. Many studies have explored the role of miRNA regulation in the immune response against bacteria. Taganov et al. [51] first described miRNA-146a. MiR-146a/b, miR-21, miR-155, and the let-7 family are the most extensively studied miRNAs in innate immune response, regulating several steps of the related pathways [52–54]. These miRNAs activate Toll-like receptors (TLRs). TLRs play crucial roles in the immune system by recognizing pathogen-associate molecular pattern (PAMP) derive from various microbes. Specifically, these and other miRNAs activate some of the components of TLR signaling, leading to regulating the extent and timing of the inflammatory response [55]. Genetic mutations in miRNAs greatly affect the development of the immune system and immune response and can lead to autoimmune, inflammatory, and cancer diseases [29]. To date, no studies have been published on the association of miR-146a polymorphism and brucellosis. We reported that at position rs2910164, co-dominant and dominant models differed significantly between the case and control groups also there was no significant difference in the alleles frequencies of rs2910164 between cases and controls. Several studies have provided evidence that there was no significant difference in the distribution of the miR-146a (rs2910164 G/C) genotypes in various diseases including TB, gastric cancer, coronary artery, and colon cancer between the patient and control groups [56–59]. A study in Brazil showed that the mutation of SNP G > C (rs2910164), and especially the C allele, was associated with susceptibility to leprosy [60]. Another study suggested that miR-146a plays an important role in rheumatoid arthritis [61]. In a study of hepatitis B patients in China, they examined the changes of miRNA polymorphisms in response to hepatitis B vaccination antibody and they found that there was a significant difference in the frequency of the position of rs2910164 between responders and non-responders, also the CC genotype resulted in a 1.74-fold increase compared to the other genotypes [62]. In Japan, Song et al. [63] found that people with rs2910164 CC genotype had a significantly increased risk of intestinal metaplasia and dysplasia and rs2910164 C allele was associated with an increased risk of intestinal metaplasia and dysplasia only among individuals infected with *Helicobacter pylori*. In our study of this situation, the frequency of GG and CC genotypes is higher in patients than in controls, while the prevalence of GC genotypes is higher in controls than in patients. In the present project, analysis of allele’s frequency of rs57095329 A/G revealed no statistically significant differences between patients, and controls and the rs57095329, co-dominant and recessive models were significantly associated between the patient and healthy groups. Shao et al. [35] in sepsis, showed that the C allele and the CC genotype were more prevalent at position rs2910164. However, no significant difference was found between allele and genotype at position rs57095329. In 2020 in the Indian population determined that the GC genotype had a significant influence on the susceptibility to colorectal cancers [64]. A previous study [65], found significant differences in the allele G and CC genotype of miR-146 C > G SNP between the pulmonary TB patients and healthy controls in the Kazak population, while in the Uygur population there were no significant differences in the frequencies of alleles and genotypes of miR-146a. The results of Damodaran et al. [66] a study of case–control in India by examining two-position rs2910164 and rs57095329 in miR-146a reported that the AG genotype at rs57095329 in the control group was significantly higher than in the patient group, while the rs2910164 position had nothing to do with the prostate cancer. In lupus, participants with G (rs57095329) allele had reduced expression of the miR-146a [36]. In contrast to our study based on the results of Zhou et al. [34], no significant difference was observed between Vogt–Koyanagi–Harada (VKH) patients in both position rs2910164 and rs57095329 However, in people with Behçet’s disease, the rs2910164 CC genotype, and C allele were significantly reduced compared to the control group. It is worth mentioning that there was a significant difference in the A C haplotype between the patients and the controls. In numerous reports demonstrated that there are no significant differences between haplotypes of rs2910164 and rs57095329 in the patient group compared to the control group [41, 67]. Cui et al. [67] showed that the rs57095329 polymorphism in the promoter region of miR-146a is involved in the risk of drug-resistant epilepsy (DRE), and the rs57095329 A allele was found to be associated with a reduced risk of seizures frequency in DRE patients. However, the rs2910164 variant was not associated with epilepsy. In AD, determined that the AA genotype of the rs57095329 polymorphism was associated with an increased risk for cognitive decline in patients [41].

## Conclusion

In the current study, we demonstrated that the dominant and co-dominant inheritance models of rs2910164 associated with brucellosis. As well as dominant and recessive models were associated with brucellosis at position rs57095329 and A C haplotype (rs2910164 and rs57095329) was associated against brucellosis. Further studies on the larger sample sizes are required to verify the observed associations. Besides, functional studies are required to identify fundamental mechanisms.

## Data Availability

The datasets used and/or analyzed during the current study available from the corresponding author on reasonable request.

## Abbreviations

T-ARMS-PCR: Tetra-primer amplification refractory mutation system-polymerase chain reaction
RFLP-PCR: Restriction fragment length polymorphism-polymerase chain reaction
SNPs: Single nucleotide polymorphisms
miRNAs: MicroRNAs
